# Insights into the role of sulfated glycans in cancer cell adhesion and migration through use of branched peptide probe

**DOI:** 10.1038/srep27174

**Published:** 2016-06-03

**Authors:** Jlenia Brunetti, Lorenzo Depau, Chiara Falciani, Mariangela Gentile, Elisabetta Mandarini, Giulia Riolo, Pietro Lupetti, Alessandro Pini, Luisa Bracci

**Affiliations:** 1 University of Siena, Department of Medical Biotechnologies, Siena 53100, Italy; 2 SetLance srl, via Fiorentina 1, Siena 53100, Italy; 3 University of Siena, Department of Life Sciences, Siena 53100, Italy

## Abstract

The tetra-branched peptide NT4 selectively binds to different human cancer cells and tissues. NT4 specifically binds to sulfated glycosaminoglycans on cancer cell membranes. Since sulfated glycosaminoglycans are involved in cancer cell interaction with the extracellular matrix, we evaluated the effect of NT4 on cancer cell adhesion and migration. We demonstrated here that the branched peptide NT4 binds sulfated glycosaminoglycans with high affinity and with preferential binding to heparan sulfate. NT4 inhibits cancer cell adhesion and migration on different proteins, without modifying cancer cell morphology or their ability to produce protrusions, but dramatically affecting the directionality and polarity of cell movement. Results obtained by taking advantage of the selective targeting of glycosaminoglycans chains by NT4, provide insights into the role of heparan sulfate proteoglycans in cancer cell adhesion and migration and suggest a determinant role of sulfated glycosaminoglycans in the control of cancer cell directional migration.

In previous papers we reported the synthesis and biological activity of stable tetra-branched peptides containing the sequence of human neurotensin (NT4), coupled with different tracers or chemotherapy drugs. NT4 peptides bind with high selectivity to cells and tissues from human cancers, such as colorectal cancer, pancreas adenocarcinoma and urinary bladder cancer, and can efficiently and selectively deliver drugs or liposomes for cancer cell imaging or therapy. By conjugating NT4 with methotrexate or 5FdU, we obtained significantly higher reduction of tumor growth in mice than in mice treated with the same amount of unconjugated drug. More recently, we found that conjugation of paclitaxel to NT4 led to increased therapeutic activity of the drug in an orthotopic model of breast cancer in mice and produced tumor regression which was not achieved with unconjugated paclitaxel in identical experimental conditions^1^,^2^,^3^,^4^,^5^,^6^. NT4 branched peptides were therefore proposed as promising selective cancer theranostics.

We found that the much higher binding of NT4 peptides than native neurotensin to cancer cell lines and human cancer surgical samples was generated by a switch in selectivity towards additional membrane receptors, which are selectively expressed by different human cancers. We demonstrated that the branched structure enables NT4 to bind membrane sulfated glycosaminoglycans (GAG), as well as different membrane endocytic receptors belonging to the low density lipoprotein receptor related (LRP) protein family such as LRP1 and LRP6, which are already known to be potentially druggable tumor markers involved in cancer biology^7^. Systematic modification of the neurotensin sequence in the NT4 peptide led to identification of a multimeric positively-charged motif that mediates interaction with heparin and endocytic receptors. The motif is very similar to heparin-binding motives contained in midkine and other proteins, like Wnt, which bind sulfated glycans and LRP receptors and are over-expressed in cancer^7^.

GAGs are large, linear, negatively charged polysaccharides consisting of repeating disaccharide units that can be sulfated at different positions and to different extents. Five glycosaminoglycan chains have been identified: heparan sulfate (HS), chondroitin sulfate (CS), dermatan sulfate (DS), and keratan sulfate and the non-sulfated hyaluronic acid^8^.

Sulfated GAG chains are linked covalently to core proteins, generating proteoglycans. Depending on the core protein, these can be divided into transmembrane (syndecan), GPI-anchored (glypican), and secreted (perlecan) heparan sulfate proteoglycans (HSPG)^9^,^10^,^11^.

The biological functions of HSPG reside in their ability to interact with various ligands, and this is strictly related to the extent to which sulfated groups of their GAG chains can be modulated. Chain structure and especially the amount and position of sulfated groups in GAGs are essential for HSPG specificity and affinity toward different ligands^12^,^13^.

Sulfated GAGs modulate cell differentiation as well as cell–cell and cell–ECM interactions by binding to several bioactive molecules, including chemokines, cytokines, growth factors, morphogens, adhesion molecules and matrix components, such as collagen, fibronectin, laminin and vitronectin^14^,^15^,^16^.

As a consequence of their specific binding to several growth factors and morphogens, sulfated GAGs are able to regulate cell differentiation and are involved in epithelial mesenchymal transition and carcinogenesis^9^,^11^,^17^. Moreover, by binding to heparin-binding sites of ECM components, sulfated GAGs collaborate with integrins for cell-ECM interactions in cell adhesion and migration^18^,^19^. Sulfated GAGs are therefore essential regulators of cancer progression through modulation of cell differentiation, invasion and metastasis.

Compared with non-neoplastic ECM, tumor associated ECM contains higher concentrations of various growth factors and large amounts of specific proteoglycans and GAGs^8^,^10^. Cancer cell membranes and tumor associated ECM are also characterized by a predominant presence of highly sulfated GAGs, which have already been identified as tumor markers in cancers such as hepatocellular carcinoma (where glypican 3 is a clinically experimented marker)^20^, breast cancer^21^, ovarian cancer^22^,^23^, colorectal cancer^24^, and others^25^. Moreover, enzymes regulating membrane shedding of HSPG as well as sulfatases, which regulate the number of sulfated groups on the GAG chain, are known to have a determinant role in cancer development and invasiveness^26^,^27^,^28^. Nonetheless, the molecular basis of the biological function of sulfated GAGs is still poorly defined, mainly due to the lack of specific HSPG ligands that could enable the role of the glycan chains to be discriminated from that of the core protein.

Endocytic receptors, like LRP1, LRP6 and sortilin, are known to bind heparin-binding ligands, such as Wnt, sclerostin, ApoE and midkine, by means of electrostatic interactions between spots of negatively and positively charged residues on receptors and ligands, respectively^29^,^30^. LRP receptors and sortilin are both known to play a role in cell adhesion, migration and differentiation^31^,^32^,^33^,^34^,^35^, though as in the case of HSPG, the molecular mechanism underlying their complex functions is far from clear.

Since NT4 branched peptides can specifically interact with sulfated GAGs and heparin-mimicking endocytic receptors, here we analyzed the effect of the tetra-branched NT4 peptide on cancer cell adhesion and motility on different supports. NT4 inhibited adhesion of PANC-1 human pancreas adenocarcinoma cells to plastic, collagen IV and oncofetal human cellular fibronectin, whereas it did not affect cell adhesion to soluble plasma fibronectin. NT4 also inhibited cancer cell migration on different supports, dramatically affecting its directionality. These results suggest that besides their possible use as selective cancer theranostics, NT4 peptides may interfere with cancer cell migration and adhesion, thus reducing tumor aggressiveness and metastatic potential. These results also provide insights into the role of HSPG and LRP receptors in cancer cell adhesion and motility on different ECM proteins.

## Results

### The tetra-branched NT4 peptide binds heparin, heparan sulfate and chondroitin sulfate with different affinity

**Figure 1:**
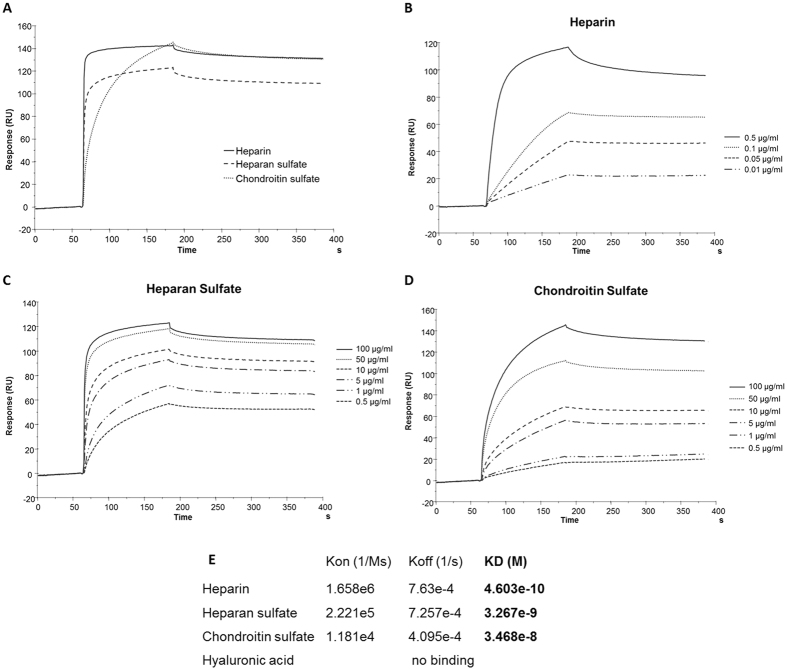
Biacore analysis of NT4-GAGs binding and affinity. (**A**) Sensorgrams of GAGs (10 μg/mL) binding to surface immobilized NT4. (**B–D**) Heparin (**B**), heparan sulfate (**C**) and chondroitin sulfate (**D**) binding to NT4. Different concentrations of GAGs were injected over biotin-conjugated NT4, previously immobilized on a SA sensor chip. (**E)** Kinetics constants and affinity values of GAGs binding to NT4 peptide.

In order to test its fine specificity, binding of the NT4 branched peptide to different GAGs was tested by Biacore. A Biotin-coupled NT4 peptide was captured on a Biacore T100 sensor chip where streptavidin had previously been covalently immobilized. Increasing concentrations of heparin, HS, CS or hyaluronic acid were then injected on the NT4-coated chip (Fig. 1). No binding of hyaluronic acid was detected. Heparin, HS and CS all bound to NT4, though with different affinity. Binding affinity of NT4 to the sulfated GAGs as measured by Biacore was higher (0.46 nM) for heparin, and decreased by about one log for HS (3.3 nM) and by another log for CS (34 nM). The highest affinity obtained with heparin, whose average number of sulfated groups is higher than that of HS and CS^12^,^13^, and the absence of binding of hyaluronic acid, which is not sulfated, suggest that sulfated groups on the glycan chains contribute to binding affinity of the branched NT4 peptide.

### Inhibition of NT4 binding to PANC-1 cancer cells by heparin, HS and CS reflects GAG-NT4 binding affinity

**Figure 2:**
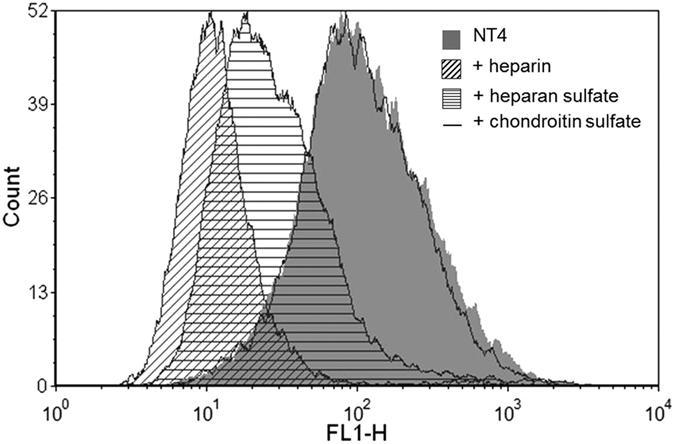
Flow cytometry of NT4 binding (grey) to PANC-1 cancer cells and inhibition by heparin (oblique lines), heparan sulfate (horizontal lines) and chondroitin sulfate (line).

Since NT4 peptide bound heparin, HS and CS with different affinity, we measured the inhibition activity of the three GAGs on NT4 binding to membrane receptors on the human pancreas adenocarcinoma cell line PANC-1 by flow cytometry (Fig. 2). Using identical concentrations of heparin, HS and CS, complete inhibition of NT4 binding was obtained with heparin, about 80% inhibition was obtained with HS and no inhibition was detected with the same concentration of CS. This result confirms the specificity of NT4 binding to membrane GAGs on cancer cells, with preferential binding to HS.

### Binding of NT4 to xylosyltransferase-I-deficient PgsA-745 cell line

The PgsA-745 cell line is derived from CHO-K1 cells treated with mutagens and screened for GAG synthesis defective mutants. PgsA-745 cells have a defect in xylosyltransferase, the enzyme responsible for coupling the first sugar in GAG synthesis, and do not produce GAGs.

**Figure 3:**
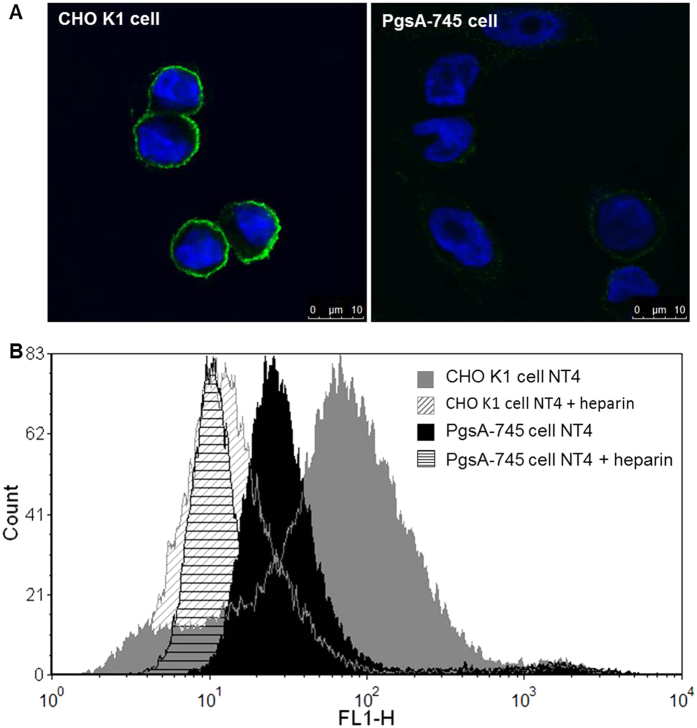
NT4 binding to CHO-K1 and PgsA-745 cells observed by (**A**) confocal microscopy and (**B**) flow cytometry. (**A)** CHO-K1 and PgsA-745 cells stained with NT4-biotin, followed by streptavidin-FITC (green). Nuclei were stained with DAPI (blue). (**B)** Inhibition of NT4 binding to CHO-K1 and PgsA-745 cells by heparin, analyzed by flow cytometry.

In order to further verify specific binding to GAGs, we compared NT4 binding to these GAG-defective cells and to native CHO-K1 cells by confocal microscopy. As expected, NT4 binding to PgsA-745 cells was much lower (Fig. 3), confirming that GAGs are specific NT4 targets on the cell membrane. Nonetheless, flow cytometry analysis demonstrated that binding of NT4 to PgsA-745 cells, though much lower than to CHO-K1, was still inhibited by heparin. Since we had already demonstrated that NT4 binds HSPG as well as endocytic receptors like sortilin, LRP1 and LRP6^7^, the residual binding to PgsA-745 cells might be due to membrane receptors belonging to these families. We had already demonstrated that heparin and endocytic receptors compete for NT4 binding.

### Inhibition of cancer cell adhesion by NT4

The results of Biacore experiments, confocal microscopy and flow cytometry described above indicate that sulfated GAGs are specifically recognized by NT4 on the cancer cell membrane. Since sulfated GAGs have an important role in cancer cell adhesion and migration, we tested the effect of NT4 on cancer cell adhesion and motility on different supports. Adhesion of PANC-1 human pancreas adenocarcinoma cells was tested on cell culture plates coated with human collagen IV, human fibronectin and on uncoated plastic wells.

Fibronectin exists in different isoforms, obtained by alternative splicing. Soluble plasma fibronectin, which is produced by hepatocytes and is abundant in plasma, lacks EDA and EDB domains^36^, whereas cellular fibronectin produced by fibroblasts, epithelial cells and other cell types is a major component of the ECM and contains the EDA and/or EDB segments, whose selective expression in cancer tissues has long been known^37^,^38^,^39^. In view of the different roles of plasma and cellular fibronectin, we tested the effect of NT4 peptide on cancer cell adhesion on both isoforms of human fibronectin.

**Figure 4:**
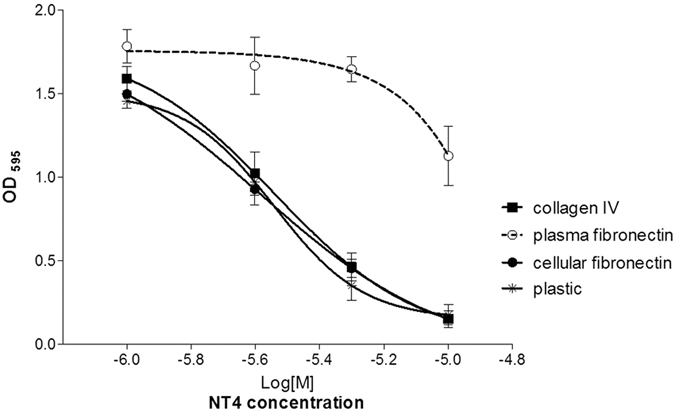
Adhesion assay of PANC-1 cells in the presence of NT4 at different concentrations on plastic, collagen IV, cellular fibronectin and plasma fibronectin. Cells were stained with crystal violet and absorbance was read at 595 nm. The data is the mean ± SD of two experiments performed in triplicate. P < 0.0001 calculated using two-way ANOVA; n (sample size) = 6.

NT4 inhibited adhesion of PANC-1 cancer cells to plastic, collagen IV and cellular oncofetal fibronectin with overlapping efficiency (EC50 of 2.8e-6 M), whereas adhesion of cancer cells to plasma fibronectin was scarcely affected and only at the highest concentration of NT4 (Fig. 4). This result suggests major involvement of HSPGs in PANC-1 cancer cell adhesion to cellular fibronectin of the ECM, compared to plasma fibronectin, and a possible role of the EDA and/or EDB domain in fibronectin binding to sulfated GAGs.

### Inhibition of cancer cell motility by NT4

**Figure 5:**
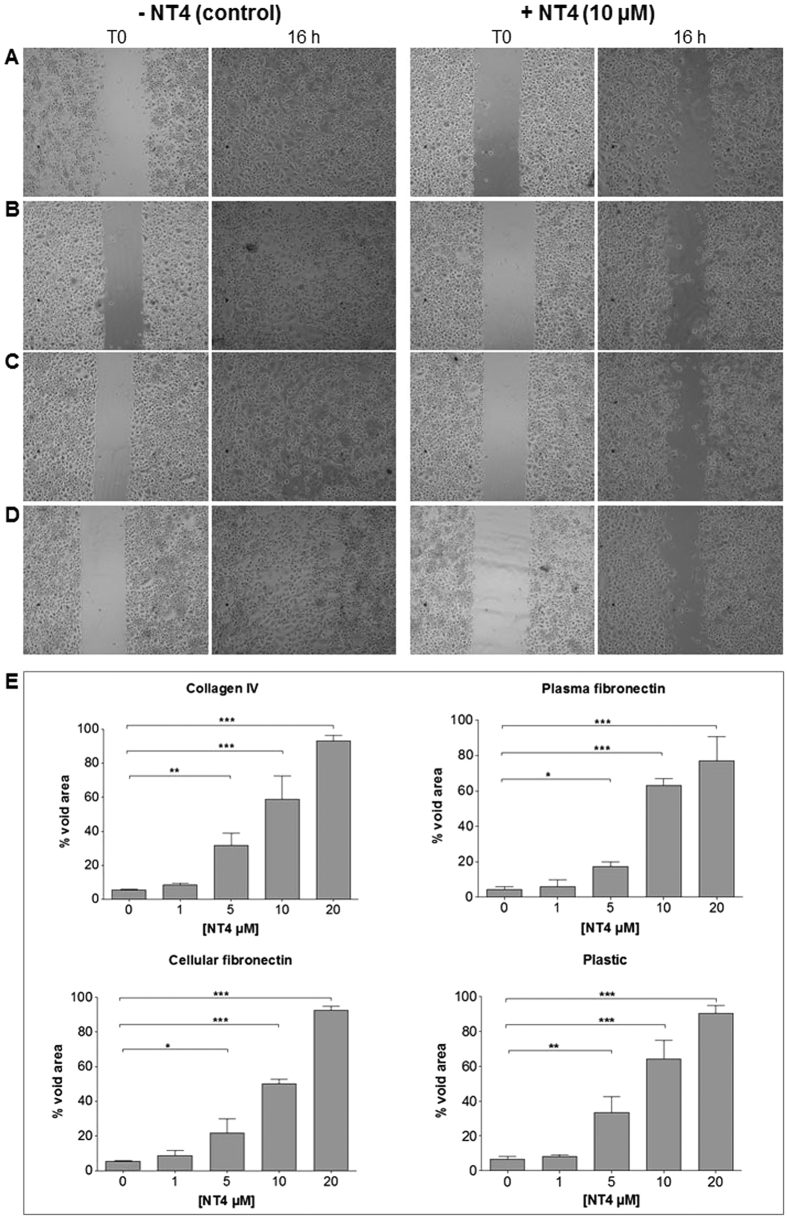
Wound healing assay. PANC-1 cancer cells were plated on wells coated with collagen IV **(A)**, plasma fibronectin **(B)**, cellular fibronectin **(C)** or on uncoated wells **(D)** where a silicon spacer had been placed immediately before cell plating. Once cells had reached confluence, the silicon spacer was removed and cells were treated with different concentrations of NT4 peptide (from 20 μM to 1 μM) for 16 hours. (**A–D**) results obtained with 10 μM NT4. Phase-contrast microscopy images were acquired from each well at time 0 and at the end of experiment (16 hours) and analyzed for recording void area, which was reported as percent of void area at time 0, for each sample (**E**). Magnification 5x. *p < 0.05, **p < 0.01, ***p < 0.001 calculated using one-way ANOVA with DUNNETT post-test; n (sample size) = 5.

The effect of NT4 peptide on cancer cell migration was tested *in vitro* in wound healing experiments. PANC-1 cells were grown to confluence on 24-well cell culture plates coated with collagen IV, cellular fibronectin or plasma fibronectin and on uncoated wells. A cell-free zone was created by inserting a silicone spacer, which was removed once cells had reached confluence. Cells were then incubated with different concentrations of NT4 and the void area was monitored. In order to distinguish cell division from cell motility, which might both interfere with filling of the void area, the effect of NT4 on cell division was checked by MTT assay. NT4 clearly inhibited filling of the cell-free area on uncoated plastic wells as well as on plates coated with collagen IV, cellular fibronectin and plasma fibronectin and the effect was dose-dependent (Fig. 5). No effect of NT4 peptides on cancer cell division was detected in identical experimental conditions (not shown), indicating that the effect on cancer cell re-colonization of the void area was entirely due to inhibition of cell migration.

**Figure 6:**
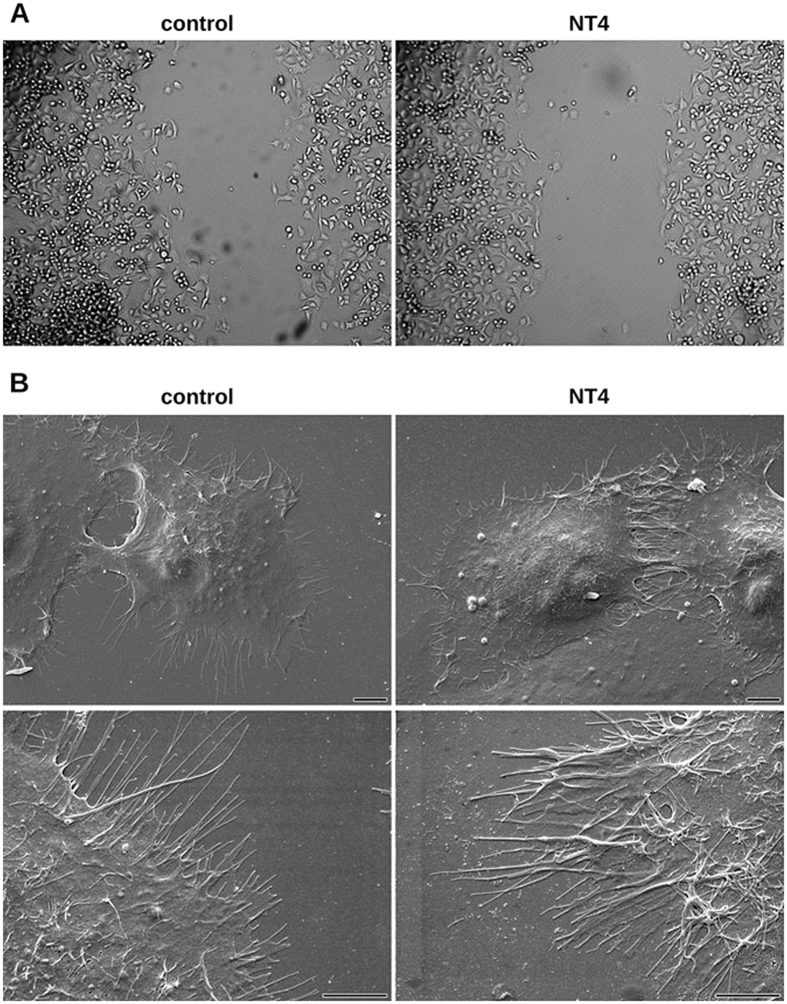
Cancer cell morphology of PANC-1 in control wells (left panels) and in the presence of 10 μM NT4 peptide (right panels) analyzed by phase contrast light microscopy in wound healing experiments (**A**) (images taken after 3-hour time lapse analysis of cell migration. Magnification 10x) and by scanning electron microscopy under static conditions (**B**). Scale bar 5 µm.

**Figure 7:**
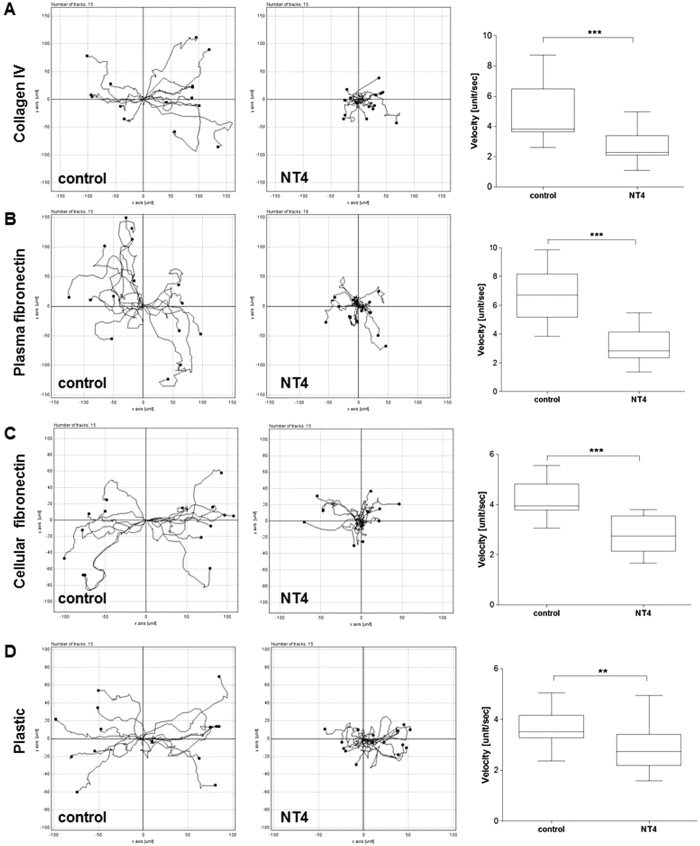
Directionality of cancer cell migration analyzed by time lapse microscopy. PANC-1 cancer cells were cultured on collagen IV **(A)**, plasma fibronectin **(B)**, cellular fibronectin **(C)** and plastic **(D),** with and without 10 μM NT4 (central panels and left panels, respectively). Cells were tracked every 20 min for 10 hours post-wounding and their paths plotted on a polar grid. Each plot represents 15 individual cell tracks. Velocity (unit/sec; where unit correspond to nm) of each analysed cell is reported in the box plot graph (right panels) where the median value is indicated by the line inside each box. **p < 0.01, ***p < 0.001 calculated using one-tailed Student t-test; n (sample size) = 15.

Cancer cell migration was monitored by time lapse microscopy in order to visualize modifications of cancer cell morphology or motility produced by the NT4 peptide. Time lapse imaging of cancer cell movements indicated that NT4 does not affect cancer cell morphology or ability to produce protrusions (Fig. 6A, supplementary material Figs S1 and S2) but rather the directionality and polarity of cell movement. In wound healing experiments on plates coated with collagen IV, fibronectins or uncoated wells, cancer cells incubated without NT4 clearly moved towards the cell-free zone, as indicated by single cell migration tracking (Fig. 7). On the other hand, when incubated with NT4, cancer cells seemed to slip on the plate and lose their direction of migration. They produced protrusions like in control wells, however these did not produce effective forward migration but rather random circular movement with little progression toward the cell-free zone (Figs 6A and 7, supplementary material Figs S1 and S2).

NT4 ability to inhibit cell migration of PANC-1 cell was also confirmed in wound healing experiments where cells were covered with Matrigel. As expected, in these experiments cell migration and speed were reduced compared to 2D wound healing (supplementary material Figs S3–S5).

The effect of NT4 on cancer cell morphology was also checked by scanning electron microscopy. PANC-1 cells were incubated with 10 μM NT4 for 3 hours and then analyzed (Fig. 6B). No modification of general cell shape or cell protrusions was observed.

### Inhibition of cancer cell trans-well migration by NT4

**Figure 8:**
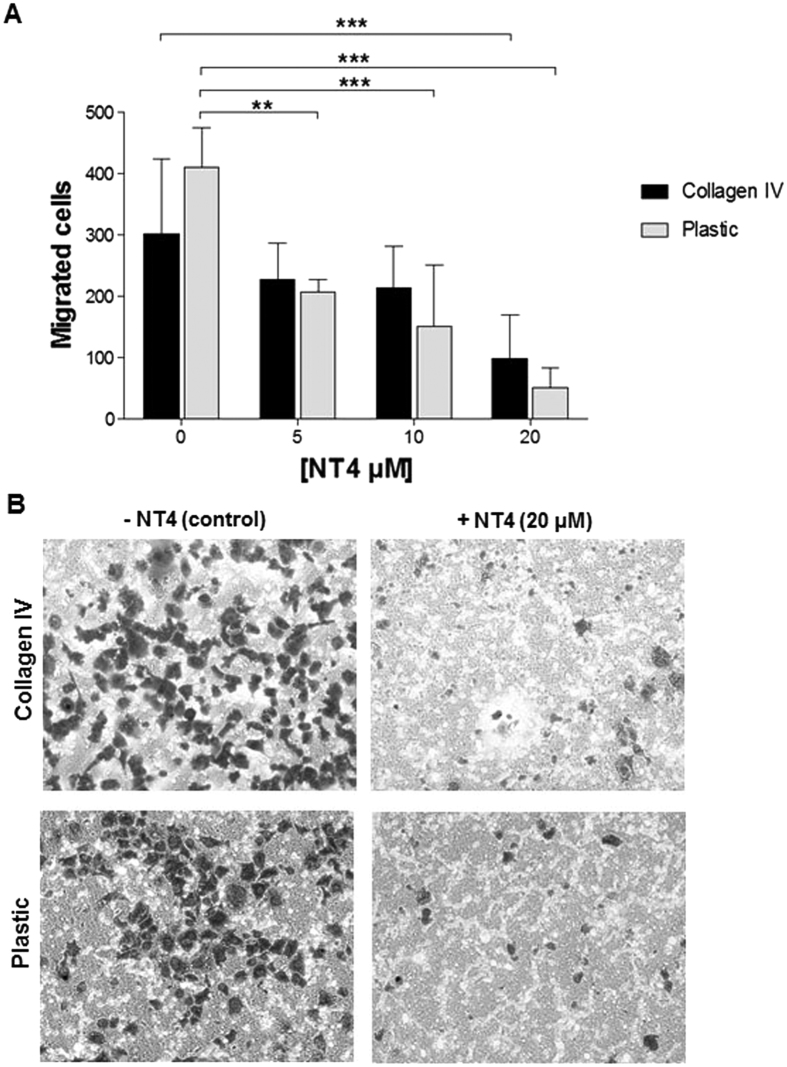
Effect of NT4 on PANC-1 cell trans-well migration in Boyden chambers. PANC-1 cells in the presence of different NT4 concentrations (20, 10 and 5 μM) were seeded on the upper component of trans-well inserts of uncoated or collagen IV–coated Boyden chambers and then incubated for 24 h. Migrating cells **(A)** were counted as described in the Methods section. (**B)** Representative images of migrated cells after 24 h, fixed and stained with Crystal violet. **p < 0.01, ***p < 0.001 calculated using one-way ANOVA with DUNNETT post-test; n (sample size) = 10.

In order to evaluate whether NT4 peptide inhibited cancer cell trans-well migration, a classical migration assay on Boyden chambers was performed with PANC-1 cells (Fig. 8). Cells were incubated for 24 h with different concentration of NT4 (20, 10 and 5 μM) in uncoated or collagen IV-coated upper wells and migrated cells were then counted. Results in Fig. 8 show that 20 μM NT4 caused about 85% and 68% reduction of PANC-1 cells trans-well migration in uncoated and in collagen IV-coated Boyden chambers, respectively.

## Discussion

In previous papers we reported that NT4 tetra-branched peptides are promising cancer-selective theranostic agents by virtue of their ability to selectively deliver different functional units to cancer cells for imaging or therapy. NT4 contains four copies of the human neurotensin sequence, which many years ago had been reported as a potential selective cancer-targeting agent because neurotensin receptors seemed to be over-expressed in different human cancers. However, we demonstrated that the high cancer selectivity of NT4, not shared by monomeric native neurotensin, is actually generated by its multimericity, which switches NT4 selectivity towards membrane receptors different from the canonical NTR1 and NTR2 G protein-coupled neurotensin receptors. NT4 was found to bind heparin, as well as LRP1, LRP6 and sortilin, by means of a multimeric positively-charged motif that might interact with negatively-charged motives in sulfated GAGs and LRP receptors, already known to share several different heparin-binding ligands^7^.

Sulfated GAGs and LRP receptors are over-expressed in different cancer tissues and are involved in several aspects of cancer cell differentiation, as well as cancer cell interaction with ECM, cell adhesion and migration^9^,^11^,^14^,^15^,^16^,^20^,^21^,^22^,^23^,^24^,^25^,^33^,^34^,^35^.

Considering the fundamental role of sulfated GAGs in many aspects of cancer cell biology, we investigated NT4 selectivity towards GAGs, as well as the effect produced by NT4 on cancer cell adhesion and migration. We found that NT4 binds heparin, HS and CS, which are all sulfated GAGs, but does not bind (non-sulfated) hyaluronic acid. Binding affinity of NT4 to sulfated GAGs, as measured by Biacore, was higher for heparin, and progressively lower for HS and CS. Inhibition of NT4 binding to human pancreas adenocarcinoma PANC-1 cells by the same sulfated GAGs was essentially in line with their binding affinity, with complete inhibition of NT4 binding obtained with heparin, less inhibition obtained with HS and no inhibition in the presence of the same concentration of CS. Since neither binding to NT4 or inhibition of NT4 binding to cancer cell membranes was detected with the non-sulfated GAG, hyaluronic acid, and heparin has a greater content of sulfated groups than HS and CS^12^,^13^,^40^, the results from affinity and inhibition activity indicate that NT4 specifically binds sulfated GAGs on the cancer cell surface, with preferential binding to HS. This result was further confirmed by the very low binding of NT4 peptides to the xylosyltransferase-I-deficient PgsA-745 cell line, which does not synthesize GAGs. Indeed, binding of NT4 to this GAG-defective cell line was much lower than to the native CHO-K1 cell line from which PgsA-745 is derived. Nonetheless, binding of NT4 to PgsA-745 cells, while low, was still completely inhibited by heparin. This may be explained by NT4 binding to LRP receptors on PgsA-745. Synthesis of LRP receptors is not affected by lack of xylosyltransferase I, which is responsible for transfer of the first xylose residue of any GAG chain to the protein core of HSPG and in fact wild type CHO-K1 and PgsA-745 cells were reported to have comparable amount of membrane LRP receptors^41^. Actually, we previously demonstrated that NT4 binds to heparin and LRP receptors via the same multimeric positively-charged motif and that binding to endocytic receptors is completely inhibited by heparin^7^.

Sulfated GAGs and LRP receptors both have an important role in many aspects of cancer cell biology, particularly aspects related to contact between cancer cells and ECM enabling cell adhesion and migration. We then tested the effect of NT4 in adhesion and migration of PANC-1 human pancreas adenocarcinoma cells on different ECM proteins. Cell adhesion and migration were tested on non-coated culture plates and plates coated with collagen IV or fibronectin. We tested the two main forms of human fibronectin: soluble plasma fibronectin, produced by hepatocytes, abundant in plasma, and lacking the EDA and EDB domains, and oncofetal cellular fibronectin, which has EDA and/or EDB domains, is produced by fibroblasts, epithelial cells and other cell types and is a major component of the ECM. Cancer cell adhesion to uncoated plastic wells, as well as to collagen IV and oncofetal fibronectin, was inhibited by NT4 with identical EC50, whereas cell adhesion to plasma fibronectin was almost unaffected by NT4. This confirms that sulfated GAGs are involved in cancer cell adhesion to different supports, including oncofetal fibronectin. On the other hand, the lack of inhibition of cancer cell adhesion to soluble fibronectin by NT4 suggests that EDA and/or EDB are essential for cell adhesion mediated by sulfated GAGs. This is in line with recent data indicating that cellular fibronectin and not plasma fibronectin is involved in epithelial mesenchymal transition^42^, cell adhesion and migration^43^ in different cancers.

In the wound healing assay, NT4 inhibited cancer cell migration on all supports, including serum and cellular fibronectin, in contrast to what we observed for cancer cell adhesion, where inhibition by NT4 only affected cancer cell adhesion to cellular and not to plasma fibronectin. PANC-1 cancer cell migration was also inhibited in wound healing experiments where cells were embedded in Matrigel, although cell migration and speed were reduced in this condition compared to the 2D wound healing experiments.

Comparison of cancer cell migration by time lapse analysis, with or without NT4, showed that inhibition of cell migration was not accompanied by any dramatic modification of cell morphology and NT4 did not affect cell ability to produce protrusions. Moreover, NT4 did not modify cell morphology even under static conditions, as verified by scanning electron microscopy of cells incubated with NT4. What appeared to be dramatically affected by NT4 was cell ability to move towards the void space by oriented migration. In the presence of NT4, cancer cells lost their direction of migration and their average speed was therefore significantly reduced. This was evident in wound healing experiments in uncoated wells, as well as on plates coated with collagen, with either cellular or plasma fibronectin, as well as in Matrigel. Moreover NT4 dramatically reduced cancer cell migration in trans-well experiments.

These results suggest that although sulfated GAGs appear to be crucial for cancer cell adhesion and migration, different mechanisms of cancer cell contact with ECM proteins, particularly with fibronectin, seem to be involved in cell migration in contrast with the static conditions of cancer cell adhesion experiments. Our results indicate that fibronectin EDA and/or EDB domains could be determinant for interaction with sulfated GAGs for cancer cell adhesion under static conditions, but not for cancer cell migration.

A fundamental role of HSPGs has already been reported in endothelial cell migration and mechanotransduction, where, in addition to regulating cell adhesion and migration speed, HSPGs seem to be important in sensing the direction of shear stress and transmitting the mechanical signal into the intracellular space. This signaling might be able to regulate the direction of cell migration^44^. Interestingly, in the same paper a different role of HSPG in cancer cell adhesion and migration was reported, which is in line with our results. Moreover, a monoclonal antibody targeting the HS chain of glypican 3 was recently reported to inhibit migration of hepatocellular carcinoma cells^45^.

Since NT4 peptides bind to sulfated GAGs and also to LRP receptors via the same multimeric ligand motif, we cannot distinguish which of the two receptor families is more involved in the effect produced by NT4 in cancer cell adhesion and migration. Nonetheless, results obtained with the GAG defective PgsA-745 cell line strongly suggest that the effect of NT4 on cancer cell migration is mostly related to its interference with the function of sulfated GAGs. Although it has long been recognized that HSPG are crucial for cell adhesion and migration, their role in respect of the better characterized function of integrins has not been fully characterized. HSPG were assigned a sort of supporting role in the organization of cell contacts with ECM and were generally considered as integrin co-receptors. In the last few years results were reported suggesting that beside the integrin-ECM contacts and related intracellular responses, HSPG are essential for the correct organization of focal adhesion, stress fibers and intracellular signaling leading to oriented cell migration^46^,^47^,^48^.

By using a selective ligand of HSPG sulfated GAG chains, we have here provided strong indications of a primary role of HSPG in cell adhesion and migration. Moreover, our results confirm the hypothesis of a synergic role of HSPG and LRP receptors, which besides sharing many morphogenic ligands and collaborating in modulating their cell signals, may also share binding sites on different ECM macromolecules and therefore collaborate in maintaining and modulating contacts and relationships between cancer cells and ECM, as well as in controlling the directionality of cancer cell migration. In conclusion, our results suggest that besides their possible use as selective cancer theranostics, NT4 peptides may interfere with cancer cell migration and adhesion, thus reducing tumor aggressiveness and metastatic potential. The results also provide insights into the role of HSPG and LRP receptors in cancer cell motility and adhesion to different ECM proteins.

## Experimental Procedures

### Peptide Synthesis

Peptides were synthesized on an automated multiple synthesizer (MultiSynTech, Germany) by standard Fmoc chemistry. Protected L-amino acids, Tentagel-resin and Fmoc4-Lys2-Lys-beta-Ala-Wang resin were purchased from Iris Biotech, Germany, the coupling reagent DIPEA (N,N-diisopropylethylamine) from Merck and HBTU (O-benzotriazole-N,N,N′,N′-tetramethyl-uronium-hexafluoro-phosphate) from MultiSynTech and DIPEA (N,N-diisopropylethylamine) from Merck. Tetra-branched peptides were synthesized either on Fmoc4-Lys2-Lys-beta-Ala-Wang resin or built using two consecutive Fmoc-Lys(Fmoc)-OH coupling steps to form the branched core on Tentagel-resin. NT4-biotin was synthesized on Tentagel resin with Fmoc-Lys(biotin)-OH as first coupling step, and Fmoc-PEG12-OH as second; Fmoc-Lys(Fmoc)-OH was then used to build the tetrameric core. Pyro-Glu-O-pentachlorophenylester (Bachem, Switzerland) was used for the last coupling step, since pyro-Glu is the N-terminal acid of the neurotensin sequence. Peptides were finally cleaved from the resin, deprotected and lyophilized.

HPLC purification was performed on a C18 Jupiter column (Phenomenex). Water with 0.1% TFA (A) and methanol (B) were used as eluents. Linear gradients over 30 min were run at flow rates of 0.8 ml/min and 4 ml/min for analytical and preparatory procedures, respectively. All compounds were also characterized on a BrukerUltraflex MALDI TOF/TOF Mass Spectrometer.

NT4 (pyELYENKPRRPYIL)4K2K-beta-Ala MS: m/z calculated for C333H519N91O81 [M+H]+: 7094.24. Found 7095.15. HPLC RT (from 80%A to 20%A) 26.63 min. Biotinylated NT4 (pyELYENKPRRPYIL)4K2K- PEG12- K(biotin)MS: m/z calculated for C373H594N96O95S [M+H]+: 7976.35. Found 7978.72. HPLC RT (from 80%A to 20%A) 26.99 min.

### SPR experiments

Experiments were performed on a Biacore T100 instrument (GE Healthcare). All materials were purchased from GE Healthcare unless otherwise specified.

#### NT4-biotin immobilization

NT4 biotin was captured on a CM5 sensor chip previously immobilized with streptavidin through standard amine coupling. Briefly, the sensor chip surface was activated with a mixture of 0.1 M 1-ethyl-3(3-dimetylaminopropyl)-carbodimide (EDC) and 0.4 M N-hydroxyl succinimide (NHS) for 7 minutes at a flow rate of 5 μl/min. Streptavidin was injected over the surface for 7 minutes and finally 1 M ethanolamine pH 8.5 was used to block any activated carboxyl groups. NT4 peptide conjugated with biotin, diluted in HBS-EP+ (Hepes 10 mM, NaCl 150 mM, 3.4 mM EDTA, 0.05% p20, pH7.4) at 30 μg/ml, was injected for 2 min at a flow rate of 10 μl/min.

#### GAGs kinetics

GAGs were diluted at concentrations ranging in HBS-EP+ and then injected over immobilized NT4 peptides. The sensor chip surface was regenerated with a short pulse of 10 mM NaOH / 0.5 M NaCl 5 minutes after the end of the injections.

Kinetics were analyzed with “Biacore T100 evaluation 1.1.1” software using the Langmuir model 1:1 for fitting curves.

### Cell lines

PANC-1 human pancreas adenocarcinoma, CHO-K1 Chinese hamster ovary cell and PgsA-745 Chinese hamster ovary cell mutant deficient in xylosyltransferase (UDP-D-xylose: serine-1,3-D-xylosyltransferase) were grown in DMEM Medium (for PANC-1 and CHO-K1) and F12 K Medium supplemented with 10% fetal bovine serum, 200 μg/ml glutamine, 100 μg/ml streptomycin and 60 μg/ml penicillin. Cell lines were purchased from ATCC (The Global Bioresource Center).

### Flow cytometry

All experiments were performed using 1 × 10^5^ PANC-1, CHO-K1 or PgsA-745 cells in 96-well U-bottom plates. For peptide binding, cells were incubated with 1 μM NT4-biotin for 30 min at room temperature; inhibition of NT4 binding by GAGs was carried out incubating cells with 1 μM biotinylated NT4 and 100 μg/ml GAGs. Cells were finally incubated with 1 μg/ml Streptavidin-FITC. All dilutions were performed in PBS, containing 5 mM EDTA and 0.5% BSA.

10000 events were analyzed in a BD FACS Calibur (Becton Dickinson, NJ USA). Results were analyzed by FCS Express 4 Plus software.

### Confocal Microscopy

PgsA-745 and CHO-K1 cells were plated at a density of 3 × 10^4^ per well in 24-well plates with cover glass slides. Samples were fixed through incubation with a PBS- (137 mM NaCl, 2 mM KCl, 10 mM Na_2_HPO_4_, 2 mM KH_2_PO_4_, pH 7.4, PBS) 4% paraformaldehyde (PFA) solution for 15 min, saturated for 30 min at 37 °C with PBS-1% bovine serum albumin (BSA) and incubated with 1 μM biotinylated peptides for 30 min at room temperature, then with 0.5 μg/ml Streptavidin-FITC for 15 min at room temperature. Samples, mounted using Fluoroshield with DAPI (Sigma Aldrich), were analyzed by confocal laser microscope (Leica TCS SP5) with 364–488 nm excitation and 458–519 nm emission filters for DAPI and FITC, respectively. All images were processed using ImageJ software (NIH).

### Cancer cell adhesion

96-well plates were coated with 20 μg/ml human collagen IV and with 10 μg/ml human cellular fibronectin and human plasma fibronectin (Sigma Aldrich) for 2 h at 37 °C and then washed three times with PBS. PANC-1 cells were plated at a concentration of 1 × 10^5^ cells/well in a 96-well plate for 30 minutes at 37 °C with different concentrations of NT4 (from 1 μM to 10 μM). Cells were then fixed with PBS - 4% PFA for 15 minutes at room temperature and stained with 0.1% crystal violet in 200 mM MES (2-(N-morpholino)ethanesulfonic acid) pH 6.0 for 1 h at room temperature. The cells were then solubilized with 10% acetic acid and the absorbance was measured at 595 nm using a microplate reader. The experiment was performed twice in triplicate.

EC50 values were calculated by non-linear regression analysis using Graph Pad Prism 5.03 software.

### Wound healing

Cell migration was measured using an *in vitro* wound healing assay. Briefly, PANC-1 cells (2 × 10^5^ cells/well) were seeded on each side of a culture insert for live cell analysis (Ibidi, Munich, Germany). Inserts were placed in wells of a pre-coated 24-well plate (20 μg/ml human collagen IV and 10 μg/ml human cellular fibronectin and human plasma fibronectin (Sigma Aldrich) for 2 h at 37 °C) and incubated at 37 °C and 5% CO_2_ to allow cells grow to confluence. Afterwards, inserts were removed with sterile tweezers to create a cell-free area of approximately 500 μm and cells were treated with NT4 peptide at different concentrations (20, 10, 5 and 1 μM) in complete medium. Cells were allowed to migrate in an appropriate incubator.

CytoSMART Lux 10x System (Lonza) was used to take a picture at time point zero and every 10 min for a total of 22 h. At the beginning and end of the time intervals the wound area was visualized under an inverted microscope (Zeiss Axiovert 200 microscopy) at 5x magnification and photographed with a Nikon ACT-1 Version 2.63 camera. The percentage of void area with respect to time 0 was determined using Tscratch software after 16 h, when control wells had completely filled the gap.

The time lapse stacks of images were also analyzed using ImageJ and the two plug-ins: Manual Tracking, and Chemotaxis and Migration Tool. Individual cells were randomly selected and tracked throughout the 10 h time period.

### Scanning electron microscopy

The morphology of cancer cells was investigated by Scanning Electron Microscopy (SEM). PANC-1 cells were plated at a density of 3 × 10^4^ per well in 24-well plates with cover glass slides and incubated with 10 μM NT4 peptide in complete medium for 3 h at 37 °C. Cells were fixed in 2.5% glutaraldehyde solution in phosphate buffer 0.1 M pH 7.2 (PB) for 2 h at 4 °C, washed in PB, postfixed in 1% OsO_4_ in PB for 30 min at 4 °C, dehydrated in ascending alcohol series, incubated for 10 min in tert-butanol, and finally freeze dried.

Afterwards, the coverslip was mounted on an aluminum stub, coated with 20 nm gold in a Balzers MED010 sputtering device, and observed in a Philips XL20 scanning electron microscope operating at an electron accelerating voltage of 10 kV.

### Trans-well cell migration

Standard trans-well inserts for 24-well plates (transparent PET membrane, 8.0 μm pore size, Corning) were equilibrated with serum free tissue culture medium for 2 h at 37 °C. In a set of experiment the upper compartment was coated with 20 μg/ml of human collagen IV. 35000 PANC-1 cells diluted in serum free medium containing NT4 (20, 10 or 5 μM), were placed in uncoated or collagen IV-coated upper chambers and the lower chambers were filled with medium containing 10% serum. After 24 h incubation at 37 °C in a humidified atmosphere with 5% CO_2_, cells were fixed with 4% PFA in PBS and stained with Crystal Violet. Non-invading cells were then removed from the top of the membrane using a cotton swab dipped in PBS, according to the manufacturer’s protocol. Ten field images for each Boyden chamber were taken with a 10x objective in a Zeiss Axiovert 200 microscope. Cells were counted using the ImageJ software, NIH.

### Cancer cell migration in Matrigel

Cell migration was measured using an *in vitro* wound healing assay in the presence of Matrigel (Corning Matrigel Basement membrane Matrix). Briefly, PANC-1 cells (2 × 10^5^ cells/well) were seeded on each side of a culture insert for live cell analysis (Ibidi, Munich, Germany). Inserts were placed in 24-well plate and incubated at 37 °C and 5% CO_2_ to allow cells grow to confluence. Inserts were removed with sterile tweezers to create a cell-free area of approximately 500 μm. Wells were covered with Matrigel for 30 min at 37 °C and then 10 μM of NT4 peptide in complete medium was added to each well. Cells were allowed to migrate in an appropriate incubator. CytoSMART Lux 10x System (Lonza) was used to take a picture at time point zero and every 7.5 min for a total of 42 h.

### Statistical analysis

All our data are parametric data calculated with Kolmogorov-Smirnov (KS) test using Graph Pad Prism 5.0 software.

P values were calculated using: two-way ANOVA for adhesion assay (Fig. 4), one-way ANOVA with Dunnett post-test for wound healing assay (Fig. 5E) and for invasion assay (Fig. 8) and one-tailed Student’s t-test for directionality of cancer cell migration analyzed by time lapse microscopy (Fig. 7) using Graph Pad Prism 5.03 software. All p values were reported in figure legends.

## Additional Information

**How to cite this article**: Brunetti, J. *et al.* Insights into the role of sulfated glycans in cancer cell adhesion and migration through use of branched peptide probe. *Sci. Rep.* **6**, 27174; doi: 10.1038/srep27174 (2016).

## Supplementary Material

Supplementary Information

Supplementary Movie S1

Supplementary Movie S2

Supplementary Movie S3

Supplementary Movie S4
