# Supplementary material for: Insights into the role of sulfated glycans in cancer cell adhesion and migration through use of branched peptide probe
Source: Sci Rep. 2016 Jun 3;6:27174. doi: 10.1038/srep27174 (PMC4891694; doi:10.1038/srep27174)
Supplement: Supplementary Information [file srep27174-s1.pdf]

Insights into the role of sulfated glycans in cancer cell adhesion and migration through use of branched peptide probe

Jlenia Brunetti,[a]* Lorenzo Depau,[a] Chiara Falciani,[b] Mariangela Gentile,[c] Elisabetta Mandarini,[a] Giulia Riolo,[a] Pietro Lupetti,[c] Alessandro Pini[a,b] and Luisa Bracci[a,b]

a University of Siena, Department of Medical Biotechnologies, Siena 53100, Italy

b SetLance srl, via Fiorentina 1, Siena 53100, Italy

c University of Siena, Department of Life Sciences, Siena 53100, Italy

* corresponding author jlenia.brunetti@unisi.it

Supplementary material

Figure S1. Movie of PANC-1 cell migration in wound healing assay without NT4 peptide (control). CytoSMART Lux 10x System was used to take pictures every 10 min for a total of 22 h.

Figure S2. Movie of PANC-1 cell migration in wound healing assay with 10 µM NT4 peptide. CytoSMART Lux 10x System was used to take pictures every 10 min for a total of 22 h.

Figure S3. Movie of PANC-1 cell migration in a Matrigel wound healing assay without NT4 peptide (control). CytoSMART Lux 10x System was used to take pictures every 7.5 min for a total of 24 h.

Figure S4. Movie of PANC-1 cell migration in a Matrigel wound healing assay with 10 µM NT4 peptide. CytoSMART Lux 10x System was used to take pictures every 7.5 min for a total of 24 h.

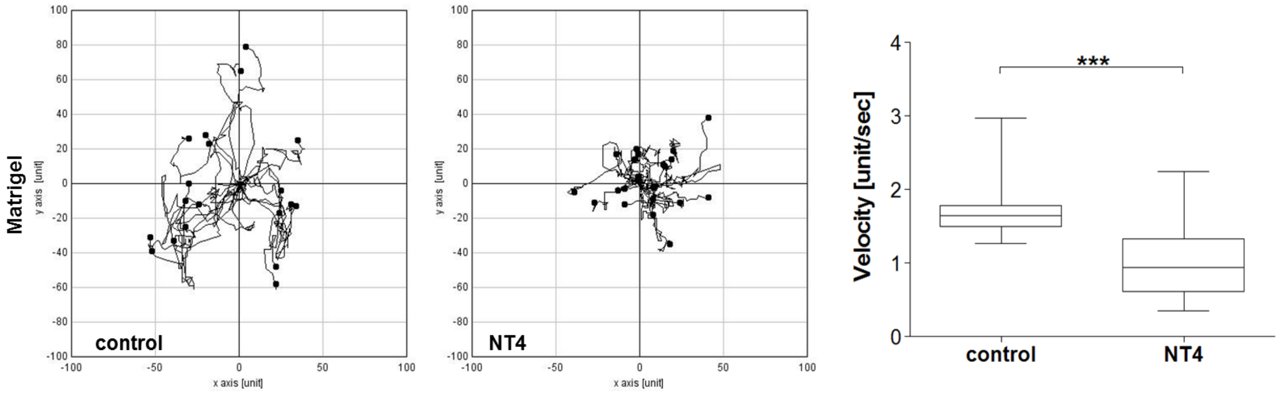

Figure S5. Directionality of cancer cell migration analyzed by time lapse microscopy using an in vitro wound healing assay in the presence of Matrigel. PANC-1 cancer cells were seeded on each side of a culture insert, covered with Matrigel and then incubated with and without 10 µM NT4 (central panels and left panels, respectively). Cells were tracked every 15 min for 10 hours post-wounding and their paths plotted on a polar grid. Each plot represents 22 individual cell tracks. Velocity (unit/sec; where unit correspond to nm) of each analysed cell is reported in the box plot graph (right panels) where the median value is indicated by the line inside each box. *** $p<0.001$ calculated using one-tailed Student t-test.
